# Mechanisms of Generating Health Disparities in the Risk of Alzheimer’s Disease

**DOI:** 10.1093/geroni/igaf122.2042

**Published:** 2025-12-31

**Authors:** Igor Akushevich, Arseniy Yashkin, Julia Kravchenko

**Affiliations:** Duke University, Durham, North Carolina, United States; Duke University, Durham, North Carolina, United States; Duke University School of Medicine, Durham, North Carolina, United States

## Abstract

Disparities in Alzheimer’s disease (AD) and related dementias (ADRD) persist across race/ethnicity, sex, and U.S. regions, yet few quantitative studies clarify how specific predictors drive these differences. Traditional methods often fall short by not addressing both the higher prevalence (exposure) and the increased risk (vulnerability) associated with a predictor. We applied two advanced approaches—the Powers-Yun decomposition technique and our recent PAF decomposition method—to quantify the exposure and vulnerability effects of each predictor using Medicare claims data from a nationally representative sample of U.S. adults aged 70, 75, 80, and 85. The analysis focused on six types of disparities: Black-White, Hispanic-White, Native American-White, Asian-White, Female-Male, and Stroke-Belt vs. non-Stroke-Belt states. Predictors included low-income status (ascertained through Medicare/Medicaid dual eligibility) and ten AD/ADRD-related diseases. We found that low income and vulnerability to arterial hypertension were the primary contributors to AD/ADRD disparities, with cerebrovascular diseases and depression as notable secondary predictors. The exposure effect dominated for income-related disparities, while hypertension’s effect was largely driven by increased vulnerability. Racial disparities (Black-White, Hispanic-White) were most affected by low income and hypertension, while Female-Male and Stroke-Belt disparities were less influenced by these predictors. Our findings indicate that different intervention strategies are needed to address AD/ADRD disparities. Low income-related disparities require targeting exposure (e.g., socioeconomic improvements), while hypertension-related disparities suggest a focus on managing vulnerability (e.g., better control of hypertension). The developed methodology offers a robust framework for explaining disparities and designing targeted interventions.

